# Portable stove use is associated with lower lung cancer mortality risk in lifetime smoky coal users

**DOI:** 10.1038/sj.bjc.6604744

**Published:** 2008-11-25

**Authors:** H D Hosgood, R Chapman, M Shen, A Blair, E Chen, T Zheng, K-M Lee, X He, Q Lan

**Affiliations:** 1 Division of Cancer Epidemiology and Genetics, Department of Health and Human Services, National Cancer Institute, National Institutes of Health, Bethesda, MD, USA; 2 Department of Epidemiology and Public Health, Yale University School of Medicine, New Haven, CT, USA; 3 College of Public Health Sciences, Chulalongkorn University, Bangkok, Thailand; 4 Department of Mathematics and Statistics, Concordia University, Montreal, Canada; 5 Chinese Center for Disease Control and Prevention, Beijing, China

**Keywords:** lung cancer, stove, mortality, fuel, home

## Abstract

Domestic fuel combustion from cooking and heating, to which about 3 billion people worldwide are exposed, is associated with increased lung cancer risk. Lung cancer incidence in Xuanwei is the highest in China, and the attributable risk of lung cancer from unvented smoky coal burning is greater than 90%. To evaluate any lung cancer mortality reduction after changing from unvented stoves to portable stoves, we used lifetime smoky coal users in a retrospective cohort of all farmers born during 1917–1951 and residing in Xuanwei in 1976. Of the 42 422 enrolled farmers, 4054 lifetime smoky coal users changed to portable stoves, 4364 did not change, and 1074 died of lung cancer. Lung cancer morality associated with stove change was assessed by product-limit survival curves and multivariate Cox regression models. Both men (*P*<0.0001) and women (*P*<0.0001) who changed to portable stoves had a significantly increased probability of survival compared with those who did not change. Portable stoves were associated with decreased risk of lung cancer mortality in male participants (hazard ratio (HR)=0.62, 95% confidence interval (CI)=0.46–0.82) and female participants (HR=0.41, 95% CI=0.29–0.57). Portable stove use is associated with reduced lung cancer mortality risk, highlighting a cost-effective intervention that could substantially benefit health in developing countries.

Globally, lung cancer is estimated to account for 1.2 million cancer deaths per year (Parkin *et al*, 2005). Approximately half of the world's population is exposed to smoke from domestic fuel used for cooking and heating, which has been classified as carcinogenic to humans (World Resources Institute, United Nations Environment Programme, United Nations Development Programme, World B, 1996; Straif *et al*, 2006). Xuanwei, China, presents a unique opportunity for investigating in-home coal smoke exposures because it has the highest prevalence of lung cancer in China and the population-attributable risk proportion of coal burning for lung cancer is over 90% (Lan and He, 2004). Lung cancer mortality rates are similar in men and women in Xuanwei, even though nearly all women and few men cook, whereas most men and nearly no women smoke tobacco (Mumford *et al*, 1987; Chapman *et al*, 1988). Indoor air pollution in Xuanwei from domestic fuel combustion for most residents is burning smoky coal (bituminous coal), with some burning smokeless coal (anthracite coal). Exposure assessments of unvented smoky coal burning in Xuanwei have found elevated levels of such carcinogens as polycyclic aromatic hydrocarbons (Mumford *et al*, 1987, 1995).

Previous reports have assessed the long-term health benefits of converting from unvented stoves to stoves with chimneys in Xuanwei smoky coal users (Lan *et al*, 2002). The benefits of portable stove use, however, have not been reported. Portable stoves are filled with coal and lighted once daily outdoors in the morning and brought indoors after visible smoke has diminished substantially and the coals are smouldering. We evaluated the effect of changing from unvented stoves to portable stoves on reducing lung cancer mortality in an extended follow-up of the Xuanwei cohort.

## Materials and methods

The study area of this retrospective cohort consists of Xuanwei's four central communes (Lai Bin, Rong Chen, Jing Wan, and Re Shui), in which most residents use smoky coal and lung cancer incidence is high (Mumford *et al*, 1987). Using local administrative records, all farmers residing in this area as of 1 January 1976, and born between 1917 and 1951, were identified. Interviews were carried out in 1992 for all subjects. Vital status of the study subjects was followed up in 1992 and 1996.

Of the 44 580 identified records, 2108 subjects were excluded because they moved from the study area, and another 50 were excluded because their identity could not be confirmed. The remaining 42 422 consenting farmers were entered into the cohort, of which 12 952 subjects underwent a stove change to portable stove (251 858.9 person-years) and 6629 did not (94 727.7 person-years). The remaining subjects underwent a stove change to a stove with chimney, which has been previously reported (Lan *et al*, 2002), or did not permanently change their stove type. Lifetime smoky coal users accounted for 4054 subjects who underwent a stove change to portable stove (78 195.9 person-years) and 4364 who did not undergo a stove change (60 322.8 person-years). Lifetime smokeless coal users accounted for 6989 subjects who underwent a stove change to portable stove (137 180.5 person-years) and 1496 who did not undergo a stove change (23 412.1 person-years). Remaining subjects were those who used more than one fuel type throughout their entire lifetime. In this report, we restricted our analyses to subjects who used the same fuel type throughout their entire lifetime. Of the 2459 lung cancer deaths identified through 1996, 1137 were in subjects who either underwent stove change to portable stove or did not undergo stove change, of which 1074 were in lifetime smoky coal users and 17 were lifetime smokeless coal users. Owing to small the sample size of lifetime smokeless coal users, analyses were restricted to only lifetime smoky coal users.

A standardised questionnaire was administered for study participants in 1992 by trained interviewers. Information was collected on demographics, type of stove used in every residence throughout life (none, firepit, portable, stove without chimney, and stove with chimney), type of fuel used in every residence throughout life (smoky coal, smokeless coal, and other), smoking history, cooking history, time spent indoors, time spent outdoors, occupations in addition to farming, residence locations, and medical history of respondent and their relatives. Dates and cause of death were extracted from death records from the four Xuanwei hospitals, including Xuanwei County Hospital, which accounted for 90% of the county's hospitalisations, public security bureaus, and public health bureaus in 1992 and in 1996 for this follow-up study. All subjects lived within 12 miles of one of the four large hospitals in Xuanwei. The hospital records of the Quijing District Hospital and Yunnan Province Hospital were also searched to ensure that information was collected on subjects who may have died outside of the county.

Each subject's age at enrolment was calculated by adding the number of days from the subject's birth date to the start of the study (1 January 1976) and dividing by 365.25 days. Each subject's follow-up time was calculated as the number of days from the start of the study to the date the subject exited the study. A subject exited the study if he or she died from lung cancer (event), died from a cause other than lung cancer before the study end (censored before 31 December 1996), or lived until the end of the study (censored by the end of study).

Product-limit survival curves were evaluated for lung cancer mortality outcome by comparing subjects who changed to portable stove with subjects who always used firepits or stoves without chimneys and underwent no change in stove type. As no difference in lung cancer incidence was evident between lifetime firepit users and those who used stoves without chimneys, the two groups were combined to serve as the reference group. Curves were constructed separately by gender, with age as the time variable. Wilcoxon, log-rank, and likelihood *χ*^2^ statistics tested across strata homogeneity.

Hazard ratios (HRs) and 95% confidence intervals (CIs) for stove change were assessed with multivariate Cox regression. Non-parametric log-rank tests of equality across strata determined if the covariate was eligible for inclusion in the final model. The appropriateness of the final Cox proportional hazard model was assessed by including time-dependent covariates, with age. If the time-dependent covariates were significant, it was assumed that the proportional hazards assumption was violated and the time-dependent variables were incorporated into the model. As the stove change variable interacted significantly with age, a time-dependent variable for change to portable stove was included that changed from zero to one at the subject's age at stove change.

The final Cox models included the time-dependent variables for change to portable stove, average tons of fuel used annually (2 to <3 tons, 3 to <4 tons, ⩾4 tons, compared with <2 tons), years of smoking (20–40 years, ⩾40 years, compared with <20 years) and years of cooking (20–40 years, ⩾40 years, compared with <20 years), along with dummy variables for history of spousal lung cancer (yes *vs* no), family history of lung cancer (parents, siblings, or children) (yes *vs* no), history of chronic bronchitis (yes *vs* no), history of tuberculosis (yes *vs* no), number of hours spent indoors until the age of 20 (<7 *vs* ⩾7 h), average number of rooms in all households throughout life (<2 rooms *vs* ⩾2 rooms), educational status (illiterate *vs* literate), and history of working as a miner (yes *vs* no). As men and women in Xuanwei have substantially different smoking (0.6% women, 90.3% men) and cooking (97.4% women, 13.6% men) prevalences, sex-specific Cox-regression models were also determined using the same covariates, as well as, history of ever cooking (ever *vs* never) in men. Birth cohort effects were controlled by stratification within models by 5-year intervals (1922–1926, 1927–1931, 1932–1936, 1937–1941, 1942–1946, and 1947–1951, compared with 1917–1921).

Sensitivity analyses were performed to assess the robustness of the study results. Sex-specific Cox regression models were restricted to only subjects where questionnaire data were provided by a surrogate, to assess differential recall bias between lung cancer cases and other study subjects. Potential biases associated with diagnosis and treatment were assessed by restricting the models to only subjects diagnosed in the provincial, district, or county hospitals, which have better resources than commune hospitals. Finally, Xuanwei is composed of 20 communes that are further subdivided into 15–20 large groups, in which extended families may cluster. Thus, sex-specific models were adjusted by large groups, which is consequently stratification by geographic regions, to evaluate the potential of familial lung cancer aggregation.

All statistical analyses were performed using SAS 9.1.3 (Cary, NC, USA). The study protocol was approved by the Chinese Academy of Preventive Medicine.

## Results

On average, subjects changed stoves at about 33 years of age and lived with the new stove for about 24 years (Table 1). Fewer subjects with portable stoves reported a history of chronic bronchitis (8.8%), emphysaema (1.8%), and tuberculosis (0.5%) than subjects with no stove change (21.5, 5.8, and 1.8%, respectively). Subjects without stove change tended to use more smoky coal over their lifetime (160.0 tons) than portable stove users (144.1 tons). Few subjects ever had jobs other than farming (22.6%). Subjects without stove change tended to have higher rates of history of lung cancer in first-degree relatives than those with portable stoves, in both men and women.

**Figure 1:**
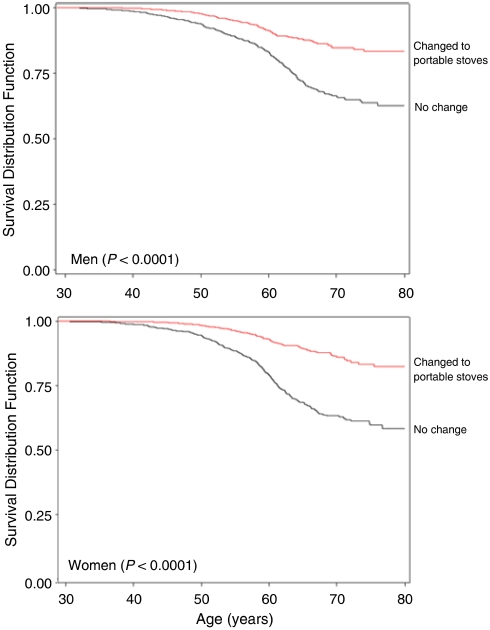
Product-limit survival curves for probability of lung cancer mortality for those who changed to a portable stove compared with those with no change.

In product-limit analysis, for both men (*P*<0.0001) and women (*P*<0.0001), the probability of dying from lung cancer was significantly lower in subjects who underwent stove change compared with those who did not (Figure 1). In both men and women, stove change was associated with a decreased risk of lung cancer mortality in lifetime smoky coal users (Table 2). Further, tons of smoky coal used annually increased risk of lung cancer mortality in a dose-dependent fashion. History of lung cancer in a spouse was also significantly associated with an increased risk of lung cancer mortality in men and women. In men, ever working as a coal miner and years of cigarette smoking were associated with an increased risk of lung cancer mortality, whereas literacy and time spending ⩾7 h per day indoors were not associated with risk of lung cancer mortality. On the contrary, in women, spending 7 or more hours indoors per day until they were 20 years old had an increased risk of lung cancer mortality, and years of cooking was associated with a nonsignificant increased risk. When restricted to only cooking years before stove change, however, the risk of lung cancer mortality associated with cooking was significantly increased in women (10–20 years: HR=3.29, 95% CI=1.58–6.83; ⩾20 years: HR=1.12, 95% CI=0.67–1.88).

Sensitivity analyses were performed to assess the robustness of the study results. When assessing recall bias, similar HRs for stove change were found when data were restricted to only subjects in which questionnaire data were provided by a surrogate (55.7%) (men: HR=0.69, 95% CI=0.49–0.96; women: HR=0.42, 95% CI=0.28–0.65). When assessing potential biases associated with diagnosis and treatment, restriction to only subjects (61.3%) diagnosed in the provincial, district, or county hospitals, which have better resources, similar results were also found (men: HR=0.62, 95% CI=0.47–0.84; women: HR=0.41, 95% CI=0.29–0.57). Finally, when assessing familial lung cancer aggregation, adjustment of our sex-specific models by large groups also yielded similar HRs of lung cancer mortality associated with stove change (men: HR=0.45, 95% CI=0.32–0.64; women: HR=0.35, 95% CI=0.23–0.52).

## Discussion

This is the first report to our knowledge to provide evidence of reduced lung cancer mortality risk associated with changing from unvented stoves to portable stoves. Our results show that portable stove use was significantly associated with decreased lung cancer mortality risk in both male and female lifetime smoky coal users. This study was performed in a geographically restricted population with minimal industrial and automotive air pollution exposures (Mumford *et al*, 1987), in which, nearly all women cook (Chapman *et al*, 1988). Even though most men smoke, similar results in stratified analyses by male participants and female participants serve to increase confidence in the reduction in lung cancer mortality observed.

As individuals who changed stoves may differ uniformly from those who did not, differences in participant characteristics may have introduced a bias. However, this bias is unlikely to explain our results. In 1976, the Chinese government sponsored a stove improvement campaign in smoky coal-using areas because of the epidemic of lung cancer. The government offered a one-time subsidy of 10 Yuan (∼$5 US at that time) to assist with stove change. By the mid-1980s, more than 80% of the residents changed stoves. Therefore, this subsidy was offered to all and accepted by a vast majority. When comparing characteristics of those who changed stoves and those who did not, we do not see much difference in the demographics (Table 1). Further, results were similar when the Cox regression models were adjusted for potential confounders including literacy, rooms in home, people in household, nationality, and other jobs besides farming. Thus, it is unlikely that our results can be explained by participant characteristics.

Although our findings strongly suggest that portable stove use is associated with a decreased risk of lung cancer mortality in smoky coal users, recall bias might have led to some spurious conclusions. For example, subjects may have unintentionally misrepresented their fuel usage throughout their lifetimes; however, as coal for heating and cooking is one of the largest expenses for families in Xuanwei, it is highly likely that they successfully recalled the amount of coal purchased. Another potential for recall bias in this study is differential recall by subjects and those for whom a surrogate responded instead. Restriction to only surrogate responders yielded similar results, suggesting that recall bias is not present. Sensitivity analyses have also ruled out major confounding and bias from differential treatment and diagnosis among cases.

The increased risk of lung cancer mortality associated, in a dose-dependent fashion, with annual tons of coal use replicates previous findings in Xuanwei (Mumford *et al*, 1987; Lan *et al*, 2002) and Shenyang (Xu *et al*, 1989, 1991). Years of cooking did not reach statistical significance in women for this analysis, which is most likely attributed to its multicolinearity with lifetime tons of fuel used as it includes fuel used for cooking, as well as heating. When restricted to only cooking years before stove change, however, the risk of lung cancer was significantly increased.

Familial lung cancer may be due to genetic predisposition or environmental exposures such as smoking and indoor air pollution. Similar to our findings, a recent meta-analysis of four cohort studies found a two-fold risk of lung cancer with a family history of lung cancer (Matakidou *et al*, 2005). We, therefore, adjusted our sex-specific models by group, which is effectively stratification by region and extended families, to determine if our observations may be explained by non-independence of familial lung cancer aggregation. This adjustment yielded similar HRs associated with stove change, suggesting that familial lung cancer aggregation is unlikely to explain our results.

Although smoking is the strongest risk factor for lung cancer globally, it was only slightly associated with increased risk of lung cancer mortality in our analyses. The relatively weak effect of smoking may result from the indoor air pollution levels in Xuanwei-overwhelming the effect of tobacco smoke. A positive but nonsignificant effect of smoking on lung cancer mortality was also observed in a cohort of elderly Chinese men (He *et al*, 2002). Further, the Asia Pacific Cohort Studies Collaboration (APCSC), involving 31 cohort studies and 480 125 subjects, found lung cancer mortality to be associated with current smoking (RR=2.48, 95% CI=1.99–3.11) in Asia and, in a more dramatic fashion, in Australia and New Zealand (RR=9.87, 95% CI=6.04–16.12) (Huxley *et al*, 2007). When comparing the results of APCSC with our study based on similar daily cigarette consumption, the APCSC found RRs similar to those of our findings associated with Asian men smoking less than 20 cigarettes per day (RR=1.35, 95% CI=1.12–1.62) and 20 cigarettes per day (RR=1.87, 95% CI=1.57–2.22) compared with non-smokers (Huxley *et al*, 2007). The nonsignificant increased risk found with smoking in our analysis supports the hypothesis that tobacco effects may be masked by the substantial levels of indoor air pollution in Xuanwei. Other factors, as yet unidentified, or the fact that our cohort is fairly young with an average exit age of 56 years, may also be relevant.

Methods of preventing harmful domestic fuel combustion exposures have focused on behavioural changes (i.e., food preparation practices and smoke avoidance), technological interventions (i.e., stove improvement, ventilation improvement, and cleaner fuel use), and various combinations of the two (von Schirnding *et al*, 2002; Barnes *et al*, 2004). Although all interventions that can potentially alleviate these harmful exposures are of public health importance, evidence suggests that behavioural changes are difficult to implement and sustain, and are less successful than home ventilation and stove improvements (Barnes *et al*, 2004). In China specifically, it has been determined that broad health risk education methods are insufficient to reduce indoor air pollution exposures from cooking and heating and that resources should be used to propagate infrastructure changes to reduce the exposures (Jin *et al*, 2006); stove improvements are, therefore, crucial for reducing the negative health effects associated with in-home fuel combustion. Stove improvements have been shown to reduce the short-term negative health effects associated with fuel combustion exposures, such as acute respiratory infections in children in India (Akunne *et al*, 2006) and eye irritation and headaches in women in Guatemala (Diaz *et al*, 2007), by reducing indoor air pollution (Naeher *et al*, 2000). Previous analyses of the Xuanwei cohort strongly suggest that stove change also reduces the long-term negative health effects associated with smoky coal combustion exposures, such as lung cancer (Lan *et al*, 2002) and chronic obstructive pulmonary disease (Chapman *et al*, 2005). These long-term health benefits result from stove interventions decreasing the indoor air pollution (Lan *et al*, 2002). The increased ventilation by adding a chimney to homes in Xuanwei reduced the average benzo(*a*)pyrene levels to 0.25 *μ*g m^−3^ compared with 1.66 *μ*g m^−3^ in unvented homes (Lan *et al*, 2002). During ignition of portable stoves, benzo(a)pyrene concentrations emitted from the portable stoves in Xuanwei have been found to be 58.33 *μ*g m^−3^ (Tain, 1996). Therefore, portable stoves allow individuals to wait until the fuel is smouldering and not have to open the stove door to add fuel many times a day as with traditional stoves, thus minimising the in-home smoke exposure.

In summary, our findings suggest that changing from unvented stoves to portable stoves is associated with a decreased risk of lung cancer mortality in both male and female lifetime smoky coal users in Xuanwei. These decreased health risks are most likely a consequence of decreased in-home smoke exposures compared with unvented homes. These results suggest that unvented homes should be upgraded to reduce the harmful exposures from solid fuel combustion, which would help to reduce the 1.6 million annual deaths attributed to indoor air pollution from solid fuel use worldwide (Ezzati and World Health Organization, 2004).

## Figures and Tables

**Table 1:** Lifetime smoky coal users by stove use history in Xuanwei, China, 1976–1996

	**No stove change (*N*=4364)**	**Change to portable stove (*N*=4054)**	**Total subjects (*N*=8418)**
*Men and women*			
Person-years	60 322.77	78 195.92	138 518.69
Age as of 1 January 1976	42.4 (±11.0)	37.2 (±10.4)	39.9 (±11.0)
Age at exit from follow-up	56.2 (±9.9)	56.2 (±9.4)	56.3 (±9.7)
Lung cancer death, 1976–1996	810 (18.6%)	264 (6.5%)	1074 (12.8%)
Died without lung cancer during follow-up	1483 (34.0%)	510 (12.6%)	1993 (23.7%)
Alive in 1996	2071 (47.5%)	3280 (80.9%)	5351 (63.6%)
Age at stove improvement	Not applicable	32.5 (±12.8)	Not applicable
Time from stove improvement until exit from follow-up	Not applicable	24.0 (±10.9)	Not applicable
People in household, first residence	4.9 (±1.7)	5.3 (±1.4)	5.1 (±1.6)
Rooms in home, first residence	1.5 (±0.8)	1.4 (±0.7)	1.4 (±0.7)
Han nationality	4206 (96.4%)	4004 (98.8%)	8210 (97.5%)
Literate	1323 (30.3%)	1759 (43.4%)	3082 (36.6%)
			
*History of*			
Chronic bronchitis	940 (21.5%)	356 (8.8%)	1296 (15.4%)
Emphysaema	253 (5.8%)	71 (1.8%)	324 (3.9%)
Tuberculosis	77 (1.8%)	19 (0.5%)	96 (1.1%)
			
People with ⩾7 waking hours indoors per day^a^	2170 (63.9%)	1237 (42.6%)	3407 (54.1%)
			
*Smoky coal*			
Tons used lifetime	160.0 (±57.8)	144.1 (±53.7)	152.3 (±56.4)
Tons used annually	3.0 (±0.9)	2.8 (±0.9)	2.9 (±0.9)
			
*Work other than farming*	694 (15.9%)	1211 (29.9%)	1905 (22.6%)
Coal miner	167 (3.8%)	18 (0.4%)	185 (2.2%)
Driver	24 (0.6%)	34 (0.8%)	58 (0.7%)
Construction worker	141 (3.2%)	407 (10.0%)	548 (6.5%)
			
*Men only* b			
Total included in analysis	2303 (52.8%)	2082 (51.4%)	4385 (52.1%)
Person-years	32 132.00	39 963.67	72 095.67
Lung cancer in first-degree relatives	140 (6.1%)	194 (9.3%)	334 (7.6%)
			
*Tobacco smoking:*			
Ever	2040 (88.6%)	1921 (92.3%)	3961 (90.3%)
Years, in ever smokers	35.2 (±10.7)	34.5 (±9.6)	34.9 (±10.2)
Age started, in ever smokers	21.2 (±3.8)	21.8 (±3.9)	21.5 (±3.9)
Ever quit, in ever smokers	35 (1.5%)	43 (2.1%)	78 (1.8%)
Filtered cigarettes per day, in ever smokers	10.8 (±7.2)	13.0 (±7.9)	11.8 (±7.6)
Non-filtered cigarettes per day, in ever smokers	13.2 (±6.0)	15.5 (±5.8)	14.5 (±6.0)
Dry pipes (cigars) per month, in ever smokers	11.7 (±6.9)	11.6 (±6.1)	11.7 (±6.6)
Water pipes per month, in ever smokers	6.7 (±4.9)	6.5 (±8.2)	6.7 (±5.7)
			
*Cooking*			
Ever	331 (14.4%)	226 (10.9%)	597 (13.6%)
Years, in ever cookers	24.7 (±13.6)	24.4 (±11.9)	24.6 (±12.8)
Age started, in ever cookers	25.3 (±14.0)	27.6 (±11.9)	26.3 (±13.1)
			
*Women only* b			
Total included in analysis	2061 (47.2%)	1972 (48.6%)	4033 (47.9%)
Person-years	28 190.76	38 232.25	66 423.01
Lung cancer in first-degree relatives	92 (4.5%)	136 (6.9%)	228 (5.7%)
Ever smoked tobacco	16 (0.8%)	6 (0.3%)	22 (0.5%)
			
* Cooking*			
Ever	1992 (96.7%)	1934 (98.1%)	3926 (97.3%)
Years, in ever cookers	34.6 (±10.4)	32.1 (±9.9)	33.4 (±10.2)
Age started, in ever cookers	16.8 (±4.4)	18.7 (±3.1)	17.8 (±4.0)

a Through age 20 years.

b Percentages by gender.

**Table 2:** Cox model HR and 95% CI for lung cancer mortality in lifetime smoky coal users in Xuanwei, China, 1976–1996^a^

	**Men and women**		**Men**		**Women**	
	**HR (95% CI)**	***P*-value**	**HR (95% CI)**	***P*-value**	**HR (95% CI)**	***P*-value**
No stove change	1.00 (reference)		1.00 (reference)		1.00 (reference)	
Stove change	0.52 (0.41–0.64)	<0.0001	0.62 (0.46–0.82)	0.001	0.41 (0.29–0.57)	<0.0001
						
Used annual average <2 tons of smoky coal	1.00 (reference)		1.00 (reference)		1.00 (reference)	
Used annual average ⩾2 to <3 tons of fuel	1.61 (1.27–2.03)	<0.0001	1.27 (0.93–1.73)	0.133	2.07 (1.43–3.00)	0.0001
Used annual average ⩾3 to <4 tons of fuel	2.19 (1.73–2.79)	<0.0001	1.77 (1.29–2.43)	0.0004	2.70 (1.85–3.93)	<0.0001
Used annual average ⩾4 tons of fuel	2.37 (1.84–3.06)	<0.0001	1.69 (1.21–2.38)	0.002	3.32 (2.24–4.92)	<0.0001
						
No lung cancer in spouse	1.00 (reference)		1.00 (reference)		1.00 (reference)	
Lung cancer in spouse	1.77 (1.43–2.18)	<0.0001	1.47 (1.07–2.02)	0.019	2.12 (1.60–2.82)	<0.0001
						
No lung cancer in parents, siblings, and children	1.00 (reference)		1.00 (reference)		1.00 (reference)	
Lung cancer in parents, siblings, and children	1.36 (1.08–1.70)	0.008	1.48 (1.10–1.99)	0.010	1.20 (0.85–1.69)	0.311
						
No history of COPD	1.00 (reference)		1.00 (reference)		1.00 (reference)	
History of COPD	1.24 (1.08–1.43)	0.003	1.33 (1.10–1.62)	0.004	1.19 (0.97–1.46)	0.103
						
No history of tuberculosis	1.00 (reference)		1.00 (reference)		1.00 (reference)	
History of tuberculosis	1.38 (0.80–2.39)	0.252	1.95 (0.96–3.95)	0.065	0.89 (0.37–2.15)	0.789
						
<7 waking hours indoors per day, until 20 years old	1.00 (reference)		1.00 (reference)		1.00 (reference)	
⩾7 waking hours indoors per day, until 20 years old	1.11 (0.98–1.26)	0.089	1.03 (0.86–1.23)	0.747	1.21 (1.02–1.45)	0.034
						
Average of <2 rooms in all houses throughout life	1.00 (reference)		1.00 (reference)		1.00 (reference)	
Average of ⩾2 rooms in all houses throughout life	1.10 (0.97–1.25)	0.155	1.18 (0.98–1.42)	0.075	1.03 (0.85–1.24)	0.755
						
Illiterate	1.00 (reference)		1.00 (reference)		1.00 (reference)	
Literate	1.19 (1.02–1.38)	0.023	1.15 (0.95–1.39)	0.146	1.27 (0.97–1.65)	0.082
						
Never coal miner	1.00 (reference)		1.00 (reference)		Not applicable	
Ever coal miner	3.34 (2.56–4.36)	<0.0001	3.39 (2.57–4.47)	<0.0001	Not applicable	
						
<20 years of smoking	1.00 (reference)		1.00 (reference)		Not applicable	
⩾20 years of pipe smoking	0.86 (0.69–1.08)	0.197	0.84 (0.64–1.11)	0.223	Not applicable	
20–40 years of cigarette smoking	1.21 (0.96–1.53)	0.114	1.22 (0.91–1.63)	0.179	Not applicable	
⩾40 years of cigarette smoking	1.42 (1.04–1.94)	0.027	1.44 (0.99–2.09)	0.054	Not applicable	
						
Never cooked	Not applicable		1.00 (reference)		Not applicable	
Ever cooked	Not applicable		0.94 (0.73–1.22)	0.650	Not applicable	
						
<20 years of cooking	1.00 (reference)		Not applicable		1.00 (reference)	
20–40 years of cooking	1.27 (1.03–1.55)	0.023	Not applicable		1.45 (0.91–2.32)	0.121
⩾40 years of cooking	1.44 (1.12–1.86)	0.004	Not applicable		1.53 (0.92–2.54)	0.103

CIs=confidence intervals; HRs=hazard ratios.

a Cox models include the time-dependent variables for change to portable stove, average tons of fuel used annually (2–<3 tons, 3–<4 tons, ⩾4 tons, compared with <2 tons), years of smoking (20–40 years, ⩾40 years, compared with <20 years), and years of cooking (20–40 years, ⩾40 years, compared with <20 years), along with dummy variables for history of spousal lung cancer (yes *vs* no), family history of lung cancer (parents, siblings, or children) (yes *vs* no), history of chronic bronchitis (yes *vs* no), history of tuberculosis (yes *vs* no), number of hours spent indoors until the age of 20 years (<7 *vs* ⩾7 h), average number of rooms in all households throughout life (<2 rooms *vs* ⩾2 rooms), educational status (illiterate *vs* literate), and history of working as a miner (yes *vs* no). Sex-specific Cox-regression models include the same covariates, as well as, history of ever cooking (ever *vs* never) in men. Birth cohort effects are controlled by stratification within the model by 5-year intervals (born during 1917–1921, 1922–1926, 1927–1931, 1932–1936, 1937–1941, 1942–1946, and 1947–1951).
